# Polymorphic Homoeolog of Key Gene of RdDM Pathway, *ARGONAUTE4_9 class* Is Associated with Pre-Harvest Sprouting in Wheat (*Triticum aestivum* L.)

**DOI:** 10.1371/journal.pone.0077009

**Published:** 2013-10-09

**Authors:** Manjit Singh, Surinder Singh, Harpinder Randhawa, Jaswinder Singh

**Affiliations:** 1 Department of Plant Science, McGill University, Quebec, Sainte-Anne-De-Bellevue, Canada; 2 Lethbridge Research Center, Agriculture and Agri-Food Canada, Lethbridge, Alberta, Canada; National Taiwan University, Taiwan

## Abstract

Resistance to pre-harvest sprouting (PHS) is an important objective for the genetic improvement of many cereal crops, including wheat. Resistance, or susceptibility, to PHS is mainly influenced by seed dormancy, a complex trait. Reduced seed dormancy is the most important aspect of seed germination on a spike prior to harvesting, but it is influenced by various environmental factors including light, temperature and abiotic stresses. The basic genetic framework of seed dormancy depends on the antagonistic action of abscisic acid (ABA) and gibberellic acid (GA) to promote dormancy and germination. Recent studies have revealed a role for epigenetic changes, predominantly histone modifications, in controlling seed dormancy. To investigate the role of DNA methylation in seed dormancy, we explored the role of *ARGONAUTE4_9* class genes in seed development and dormancy in wheat. Our results indicate that the two wheat *AGO4_9* class genes *i.e. AGO802* and *AGO804* map to chromosomes 3S and 1S are preferentially expressed in the embryos of developing seeds. Differential expressions of *AGO802-B* in the embryos of PHS resistant and susceptible varieties also relates with DNA polymorphism in various wheat varieties due to an insertion of a SINE-like element into this gene. DNA methylation patterns of the embryonic tissue from six PHS resistant and susceptible varieties demonstrate a correlation with this polymorphism. These results suggest a possible role for *AGO802-B* in seed dormancy and PHS resistance through the modulation of DNA methylation.

## Introduction

ARGONAUTE (AGO) proteins form a large family that regulates gene expression through transcriptional and post-transcriptional gene silencing; they are reviewed in [1]. In addition to their canonical role in heterochromatin silencing, recently, the involvement of the AGO4_9 class of *AGO* genes have been shown in the embryo as well as in reproductive and seed development [2]–[4]. The differential expression of *AGO1003*, an AGO4_9 class of Argonaute, in embryos of dormant vs non-dormant genotypes in barley provides further evidence of its possible role in maintaining the quiescent state of seeds, commonly known as seed dormancy [5]. Reduced seed dormancy is the most important aspect of seed germination on a spike prior to harvesting, or better known as pre-harvest sprouting (PHS).

In Canada, PHS in spring wheat causes significant economic losses due to a reduction in grain yield, end-use quality and viability of the seed for planting. The annual cost of PHS to the Canadian wheat industry is estimated at $100 million [6]. Seed dormancy is a complex trait which has a large component of genotype X environmental interactions, which are reviewed in [7]–[8]. The balance of the antagonistic action of abscisic acid (ABA) and gibberellic acid (GA) in promoting dormancy and germination constitutes the basic genetic framework of seed dormancy; nevertheless, it is influenced by various environmental factors including light, temperature and abiotic stresses [9]–[11].

Numerous studies have been conducted to understand the genetic control of seed dormancy and PHS. Several QTLs have been reported in different wheat populations since 1993; the majority are located on chromosome 3 and 4 [12]–[14]. Screening of mutants also contributes to our knowledge of ABA sensitivity and ultimately, PHS resistance in wheat [15]–[17] and barley [18].

There is increasing evidence that epigenetic modification can also influence seed dormancy. Recently, Zheng et al. [19] demonstrated that histone methylation plays an important role in establishing seed dormancy. Knockout mutants for the histone methyltransferase KYP/SUVH4, which mediates H3K9 dimethylation, exhibit increased dormancy; plants which overexpress KYP/SUVH4 show reduced seed dormancy [19]. It was further shown by Zheng et al. [19], that *KYP/SUVH4* is regulated by ABA and GA. Sensitivity of seed germination to ABA and paclobutrazol, an inhibitor of GA synthesis, is enhanced slightly in the mutant. Evidence of the role of chromatin modifiers in seed dormancy was further proven by Liu et al. [20] through cloning of the *REDUCED DORMANCY 4 (RDO4)* locus in Arabidopsis. *RDO4* encodes a C3HC4 RING finger E3 ligase that catalyzes histone H2B monoubiquitination and therefore, it was renamed *HISTONE MONOUBIQUITINATION1 (HUB1)*. Histone H2B monoubiquitination is associated with the activation of gene transcription whereas deubiquitination is linked to repression [21]. Consistent with this role, expression of several dormancy-related genes involved in ABA metabolism and signalling, such as *NCED9* and *ABI4,* are reduced in freshly harvested seeds of *hub1* mutants as compared to wild-type plants. Apart from these two studies, there is also a large body of evidence concluding the indirect influence of epigenetic modification on seed dormancy. ABA, which plays a central role in the induction of dormancy, is able to induce chromatin modification and is itself regulated by chromatin modification. ABA has been shown to induce acetylation of H3-K14 and methylation of H3-K4 [22]. Histone deacetylation is also known to affect seed germination through ABA induced gene expression [23]–[25].

Evidence for the role of DNA methylation in seed dormancy has been suggested previously. H3K9 methylation mediated by SUVH4/KYP and SUVH5, another histone methyltransferases, is also required for the maintenance of CNG methylation by the CMT3 DNA methyltransferase [26]–[27]. ARGONAUTE (AGO) proteins of the AGO4_9 class are key players in DNA silencing in plants through RNA dependant DNA methylation (RdDM; this was reviewed in [1]). Our recent investigation of *AGO1003*, an AGO4_9 class of AGO genes in barley embryos, indicates that these genes are differentially expressed in dormant and non-dormant genotypes, where the expression of *AGO1003* is significantly reduced in embryos of dormant varieties [5]. Altogether, in this study, we have explored AGO4_9 class genes from wheat, and conducted an expression analysis of *AGO802* and *AGO804* in developing embryos of wheat. Expression of *AGO802* homeolog AGO802-B was also investigated in embryos of PHS resistant and susceptible varieties of wheat. Further, we analyzed the DNA methylation status in embryos of PHS resistant and susceptible varieties.

## Results

### Identification of *AGO4_9* wheat homoeologs

It has been previously suggested that there are two *AGO4_9* class genes in barley, a diploid species closely related to wheat [5]. Thus, bread wheat, a closely related allohexaploid species with three subgenomes, is expected to have three homoeolog genes corresponding to each barley homolog. Sequence information of one *AGO4_9* homoeolog *AGO802* corresponding to the barley homolog, *AGO1003*, was previously available in the chromatin database ChromDB (www.chromdb). To obtain sequences of the remaining five homoeologs, we performed BLAST searches in three different wheat EST databases i.e. Graingenes (http://wheat.pw.usda.gov/GG2/index.shtml), JCVI (http://www.jcvi.org/), TGI (http://compbio.dfci.harvard.edu/tgi/software/) and PGDB (http://www.pygresql.org/pgdb.html). Through BLAST search utilizing the *AGO802* sequence, we obtained forty-five singleton ESTs or contigs assembled from ESTs and mRNA sequences corresponding to the wheat *AGO4_9* genes. These forty-five sequences were assembled into six distinct contigs which represented the six homoeologs in wheat corresponding to the two barley *AGO4_9* genes. For 3 of these assembled mRNA sequences, the complete 3′ UTR sequences including the polyA tail were obtained. For the remaining two genes corresponding to homologs of *AGO1003* and homoeologs of *AGO802,* we performed a 3′ RACE with gene-specific primers designed from *AGO802* and related sequences on cDNA synthesized from embryos 10 DAP. Two distinct sequences were obtained through 3′ RACE which are homologous to *AGO802*. Thus, utilizing the sequences obtained from different databases and 3′ RACE, we were able to assemble the six transcripts of wheat *AGO4_9* genes. Three of these sequences are homologous to the barley *AGO1002* gene and thus represent the three wheat homoelogs of this gene. The remaining three sequences which include *AGO802* are the wheat homoeologs of the barley *AGO1003* gene. In addition to the ESTs assembled in six homoeologs, we identified an EST which probably is a paralog of the wheat *AGO804* gene. Two more ESTs were identified which are the splice variants of an *AGO802* homoeolog.

### Phylogenetic analysis of *AGO4_9* genes

**Figure 1:**
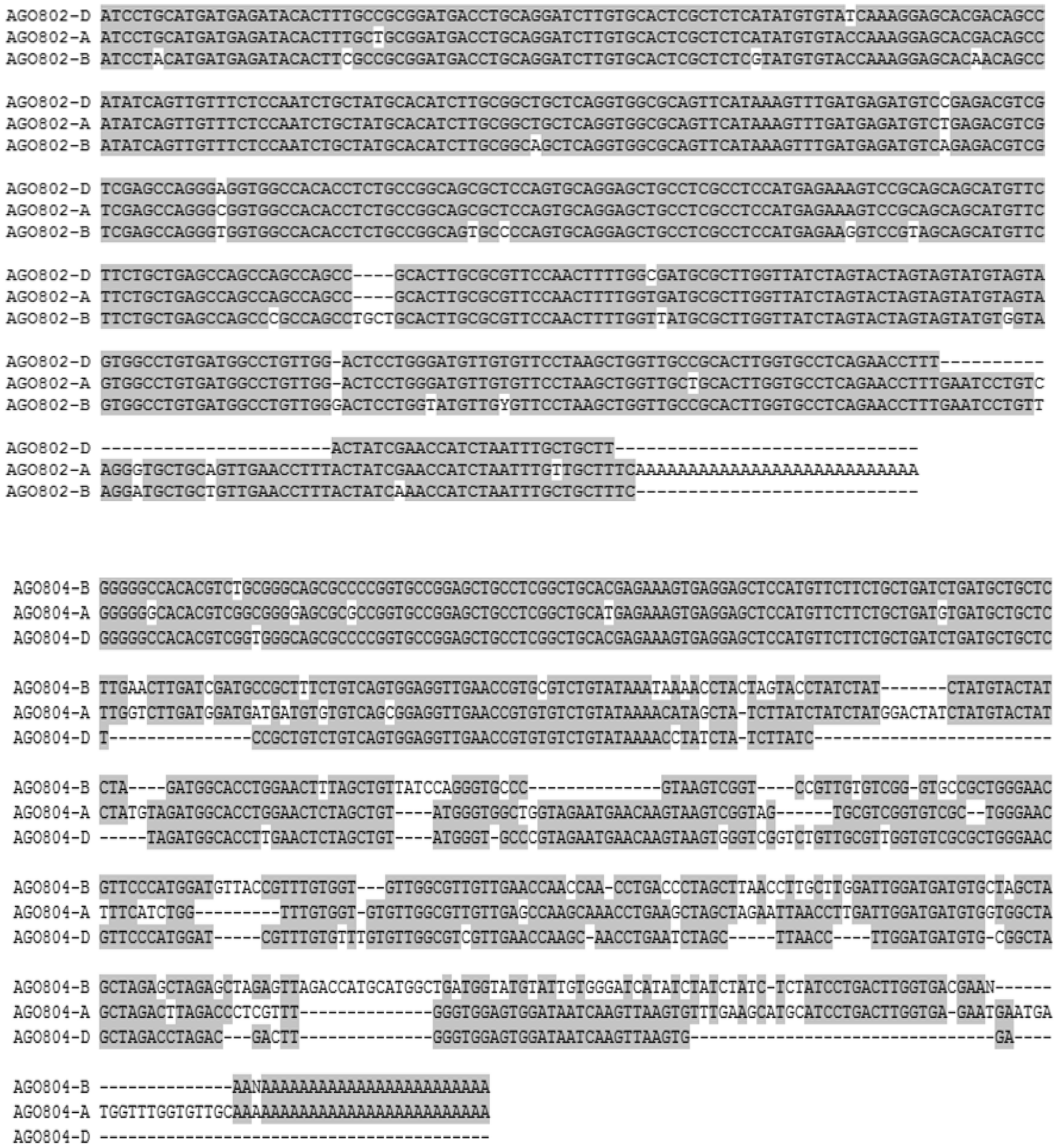
Comparing wheat homoeologs. DNA sequence comparison of wheat homoeologs of *AGO802* and *AGO804*. The three wheat *AGO802* homoeologs are almost 100% identical for the sequence and *AGO804* has sequence homology of about 98% among its three homoeologs.

**Figure 2:**
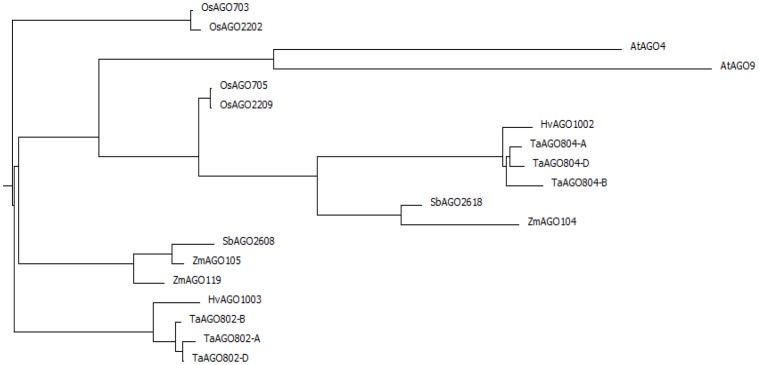
Phylogenetic comparison of ARGONAUTE4_9. Comparison of ARGONAUTE4_9 class of genes using a Phylogenetic tree from wheat, barley, rice, maize, sorghum and Arabidopsis.

Plant *Argonaute* genes are divided into three clades based on sequence and functional homology with Arabidopsis AGO proteins. AGO4_9 clade, which consists of the Arabidopsis AGO4 and AGO9 function in the DNA silencing pathway. Phylogenetic analysis of wheat AGO4_9 Argonautes based 224 aa partial protein sequences including the PIWI domain, grouped the wheat homoeologs of AGO802 and AGO804 into two distinct groups with the expected closest homology to the two barley AGOs. The three wheat *AGO802* homoeologs are almost identical for the sequence compared with the B genome homoeolog differing by only one amino acid from A and D genomes. The *AGO802* homoeologs have a 97% amino acid identity to the barley AGO1003. The DNA sequence homology for this region and the 3′UTR between the wheat homoeologs is 95–97% (Fig. 1). In contrast the sequence homology with *AGO1003* is 85%. The *AGO804* homoeologs have 98–99% amino acid identity with each other and to the barley *AGO1002*. The DNA sequence homology is 91–93% among the *AGO804* homoeologs and 81% to *AGO1002* of barley (Fig. 1). When compared with AGO4_9 proteins from other cereals, wheat AGO804 homoeologs and AGO1002 clustered in the AGO group which includes maize AGO104 and sorghum AGO2618 (Fig. 2). AGO802 homoeologs and AGO1003 cluster into a separate group distant to AGO804 and AGO1002, though are more closely related to maize AGO105 and AGO2608.

### *AGO802* and *AGO804* map to chromosome 3S and 1S

**Figure 3:**
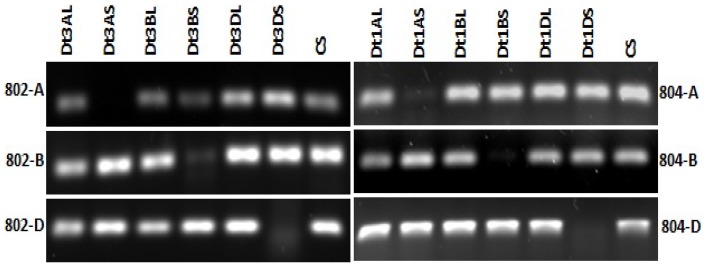
Mapping of *AGO802* and *AGO804* in wheat. Absence of amplification in lanes Dt3AS, Dt3BS and Dt3DS in AGO802, suggest its map location on chromosome arm 3S. While in AGO804, absence of amplification in Dt1AS, Dt1BS and Dt1DS lanes, confirms its map location on chromosome 1S.

The six wheat AGO4_9 genes were assigned to the specific chromosome arms of wheat utilizing the Chinese Spring nullisomic-tetrasomic lines and ditelosomic lines [28]. Gene-specific primers were designed from the 3′ UTR sequences to discriminate between the homoeologs of *AGO802* and *AGO804*. Initial PCR was performed on nullisomic-tetrasomic lines to assign the genes to a specific chromosome. Second PCR was then performed on a subset of ditelosomic lines specific to the chromosome assigned through screening of nullisomic lines. *AGO802* was assigned to chromosome 3S and the gene-specific primers were able to discriminate the three homoeologs for A, B and D genome for this gene (Fig. 3). Similarly *AGO804* was assigned to chromosome 1S and the gene-specific primers were able to discriminate the A, B and D genome homoeologs for this gene (Fig. 3).

### Expression of *AGO802* and *AGO804* homoeologs during seed development

**Figure 4:**
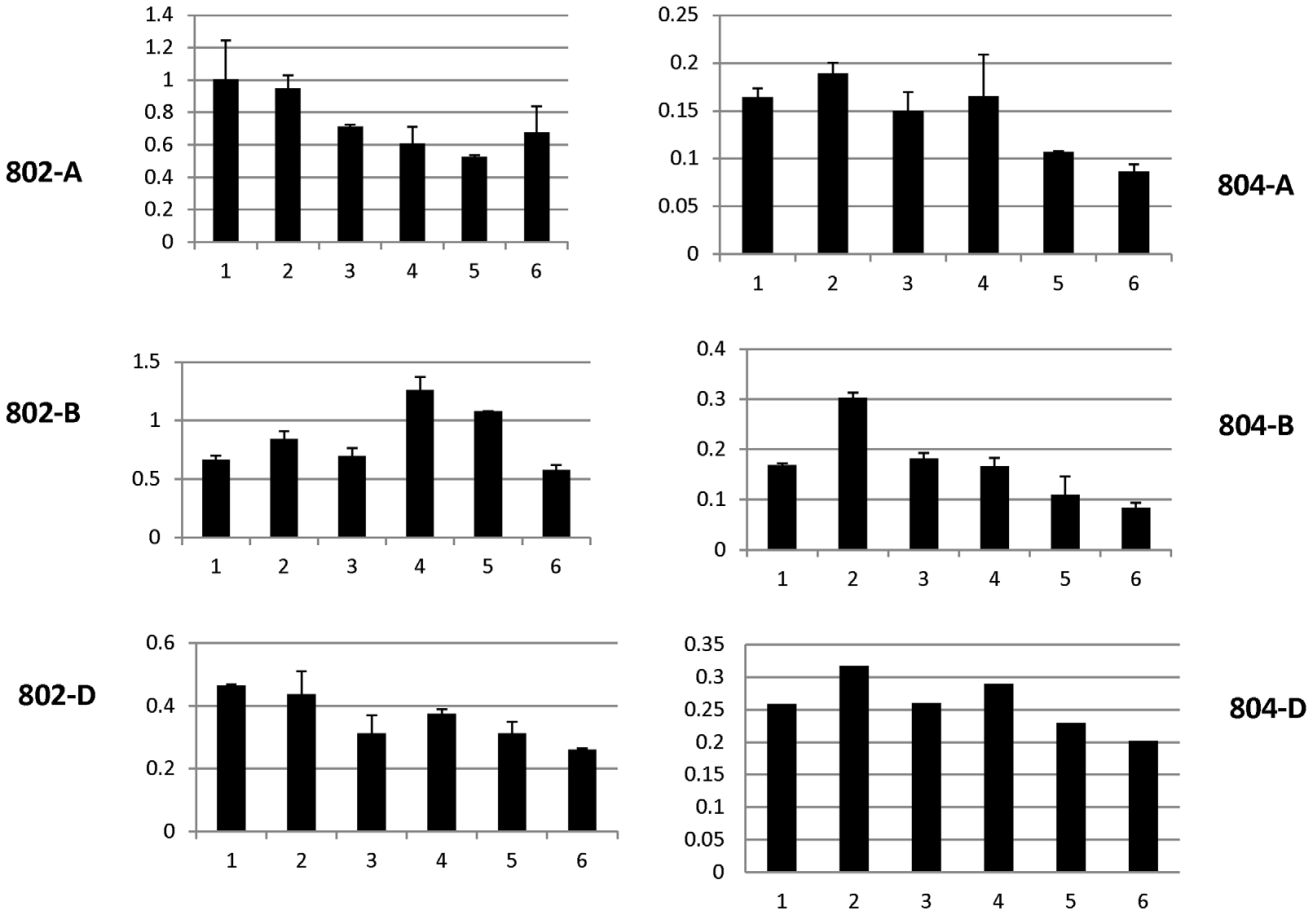
Expression analysis of *AGO802* and *AGO804*. Expression of *AGO802* and *AGO804* homoeologs in embryos during seed development in Chinese Spring cultivar. QRT-expression analysis was performed at six different stages consisting of ovaries at 5 DAP stages and embryos at 10, 15, 20, 25 and 30 DAP, shown on X-axis of graph as 1–6 respectively.

To study the expression of six wheat *AGO4_9* genes in the developing seeds, homoeolog-specific primers described for mapping were used. QRT-expression analysis was performed at six different stages consisting of ovaries at 5 DAP stages and embryos at 10, 15, 20, 25 and 30 DAP (Fig. 4). The expression patterns varied amongst the homeologs for both of these genes. Overall the expression of *AGO802* was 2–3 times higher than *AGO804* in these tissues. Amongst the *AGO802* homoeologs *AGO802A* and *AGO802B* showed higher expression than *AGO802D* albeit in different stages. *AGO802A* showed the highest expression at 5 and 10 DAP decreasing in subsequent stages of embryo development. *AGO802B* expression is lower at 5 DAP but subsequently increases to a maximum level at 20 DAP. Between 20 and 30 DAP, *AGO802B* expression decreases dramatically to less than half the level at 20 DAP. Although the expression of *AGO802D* was lower, compared to *AGO802A*, the expression pattern is similar being highest at 5 DAP and decreasing in subsequent stages. Amongst the *AGO804* homoeologs, *AGO804D* has the highest expression while *AGO804A* has the lowest. *AGO804B* has an intermediate level of expression compared to its counter-homoeologs.

### *AGO802B* gene shows polymorphism for retrotransposon insertion in Canadian wheat varieties

**Figure 5:**
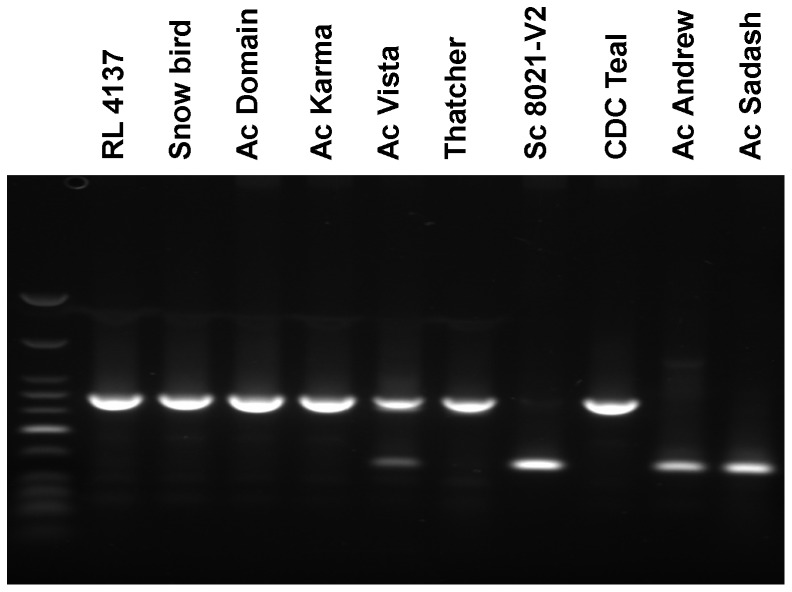
Analysis of a retro-transposon insertion in the *AGO802B* transcripts. Polymorphism as observed on agarose gel in different wheat varieties with varying reaction to pre-harvest sprouting, due to insertion of a retro-transposon insertion in the *AGO802B* transcripts.

Previous studies in our laboratory have shown that *AGO1003* is differentially expressed in the 20 DAP embryos of pre-harvest sprouting (PHS) resistant and susceptible barley varieties [5]. The expression of this gene in PHS resistant varieties was significantly lower than in PHS susceptible genotypes. To test this expression pattern in wheat, we analyzed the transcripts of *AGO802B* in ten Canadian wheat varieties. Interestingly, we observed polymorphism in the length of the fragments amplified from the genomic DNA of these varieties. Seven of the ten wheat varieties analyzed contained an insertion of 160 bp in the 3′ UTR which is inserted 30 base pairs downstream of the stop codon (Fig. 5; Table. 1). This polymorphism was also detected in the transcripts amplified through RT-PCR suggesting that the retrotransposon insertion is a stable part of the 3′ UTR and is not spliced from the mRNA. Although the BLAST analysis did not show homology to any known sequence, a nine base duplication is present at both ends of this sequence suggesting a retroelement insertion. This element likely represents a short, interspersed nuclear element (SINE). This insertion is present in the genotypes RL4137, Snowbird, AC Domain, AC Karma, AC Vista, Thatcher and CDC Teal (Table 1, Figure 5). It was absent in AC Andrew, Sadash and SC 8021-V2. With the exception of SC 8021-V2, the retro-element insertion is present in PHS resistant/tolerant varieties and is absent in PHS-susceptible varieties suggesting its correlation with the PHS trait in the wheat varieties tested. CDC Teal, a variety with a medium PHS reaction, also carried this insertion.

**Table 1:** Retroposon insertion in the *AGO802B* gene, in 10 wheat accession with varying reaction to pre-harvest sprouting.

Variety	Type	Insertion	PHS reaction
RL4137	Red Spring Wheat	Yes	Resistant
Snowbird	White Spring Wheat	Yes	Resistant
AC Domain	Red Spring Wheat	Yes	Resistant
AC Karma	White Spring Wheat	Yes	Resistant
AC Vista	White Spring Wheat	Yes	Resistant
Thatcher	Red Spring Wheat	Yes	Tolerant
SC8021-V2	White Spring Wheat	No	Resistant
CDC Teal	Red Spring Wheat	Yes	Medium
AC Andrew	White Spring Wheat	No	Susceptible
AC Sadash	White Spring Wheat	No	Susceptible

### PHS-susceptible and resistant wheat varieties are differentially methylated in 5S rDNA repeats

**Figure 6:**
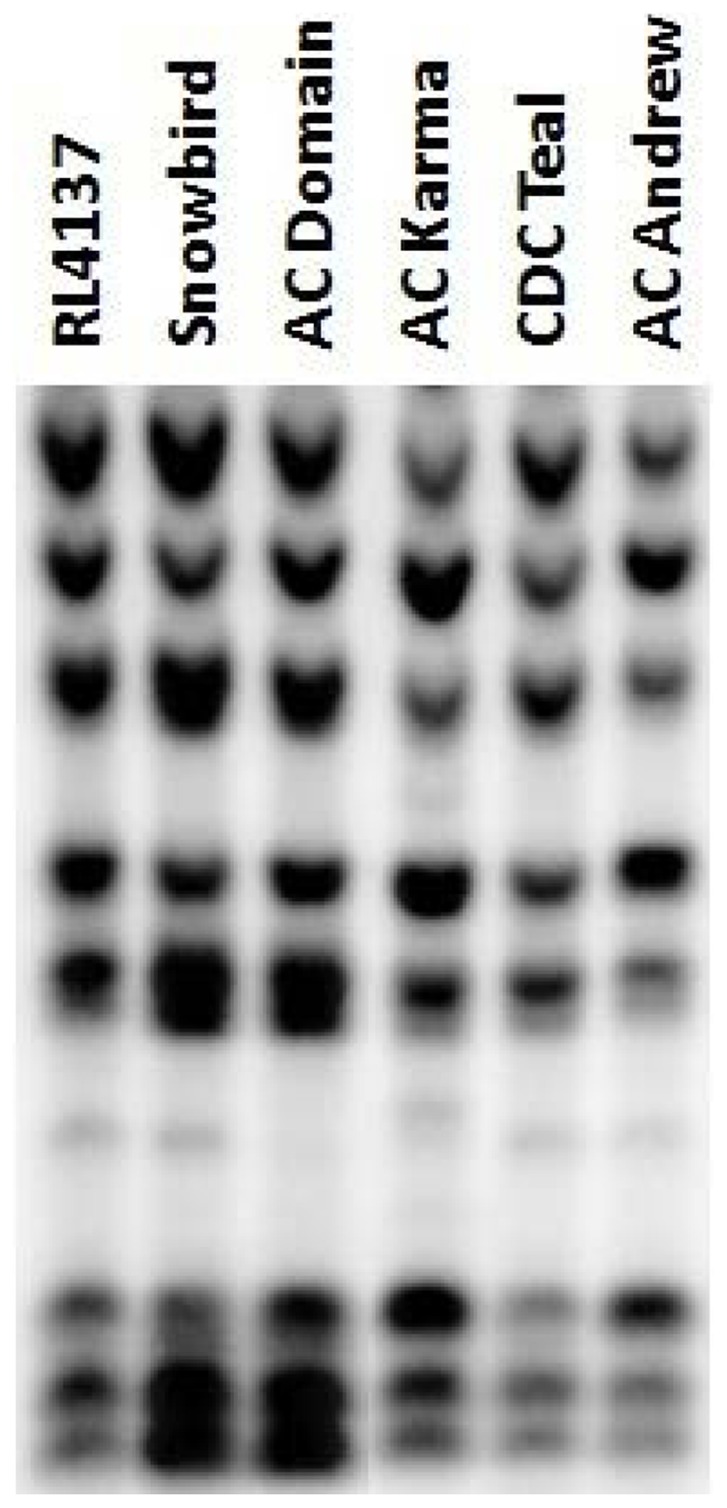
Methylation differences between wheat varieties. DNA extracted from the embryos of mature seeds was digested with *Msp* I restriction enzyme and probed with 5S rDNA. PHS resistant varieties RL4137, Snowbird, AC Domain, AC Karma show reduced methylation as compared to a PHS susceptible variety AC Andrew. DNA methylation in CDC Teal, a variety with medium PHS reaction was also less, compared to AC Andrew but more than the PHS resistant varieties.

**Figure 7:**
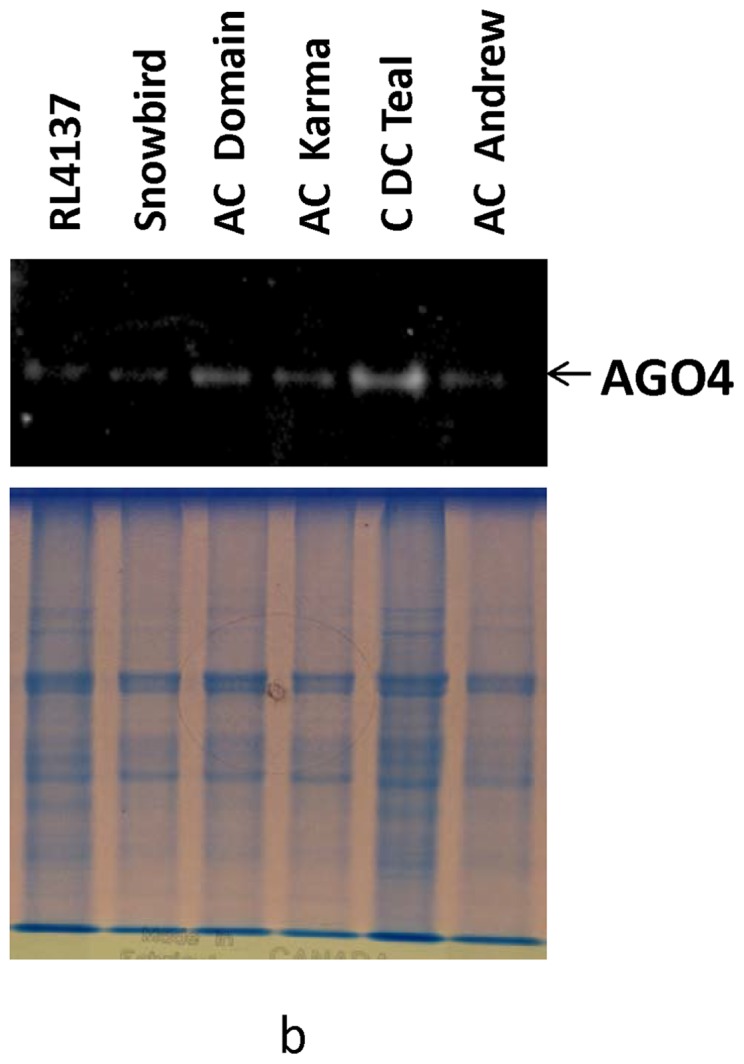
The AGO4 proteins in different wheat varieties. Total proteins from the embryos of mature seed were extracted and resolved on SDS-PAGE. Argonaute 4 antibodies detected AGO4 proteins in PHS resistant and susceptible varieties. Differential protein concentration was observed in resistant RL4137, Snowbird, AC Domain, AC Karma and susceptible varieties (CDC Teal and AC Andrew). Immunoblot and protein loading lanes correspond to coomassie gel.

Argonaute proteins of AGO4_9 clade are components of DNA methylation pathways in plants with an important role in silencing repetitive DNA and transposons. Mutants of the Arabidopsis *AGO4* and maize *AGO104* genes exhibit reduced DNA methylation in 5S and centromeric repeats [3], [29]–[30]. To test if the wheat varieties with a retro-transposon insertion in the *AGO802B* genes and variable PHS reaction, exhibit any differences in methylation of repetitive DNA, we analyzed the non-CG methylation of 5S rDNA repeats using DNA Blot analysis. The DNA from embryos of mature seeds was analyzed due to the embryo-preferential expression of *AGO802B* and the critical role of embryos in determining the PHS reaction of genotypes. DNA from six wheat varieties, namely, RL4137, Snowbird, AC Domain, AC Karma, CDC Teal and AC Andrew, was digested with methylation sensitive restriction enzyme *Msp* I and probed with 5S rDNA (Fig. 6a). The six wheat varieties showed a variable pattern for non-CG DNA methylation at 5S rDNA repeats. PHS resistant varieties RL4137, Snowbird, AC Domain, AC Karma show reduced methylation as compared to AC Andrew, a PHS susceptible variety. DNA methylation in CDC Teal, a variety with medium PHS reaction, was also less when compared to AC Andrew but more than the PHS resistant varieties. Among the four PHS resistant varieties, Snowbird and AC Domain showed the least DNA methylation. Similarly, most of PHS resistant varieties show low level of AGO 4 protein concentration while comparing with susceptible varieties (Figure 7).

## Discussion

### Wheat homologs of *AGO4_9* clade

Bread wheat (*Triticum aestivum*), an allohexaploid with three different genomes (A, B and D), arose as a result of two polyploidization events involving three diploid species. An ancient ployploidization event between the ancestral species of *Triticum monococum* and *Aegilops speltoides* created *Triticum dicocum* a tetraploid species which hybridized to *Aegilops taischii* in a more recent hybridization event to produce hexaploid bread wheat [31]. Barley, a diploid species closely related to wheat, has two genes of the *AGO4_9* clade [5]. Utilizing the sequence information from barley *AGO1002* and *AGO1003* genes and wheat *AGO802* gene homoeologs, *AGO4_9 class* genes were identified. *AGO802* is homologous to *AGO1003* and *AGO804* is homologous to *AGO1002*. *AGO802* maps to chromosome 3S whereas *AGO804* maps to chromosome 1S based on the homoeolog-specific PCR primers. DNA and protein sequence comparisons with AGO4_9 *class*genes of related cereals and Arabidopsis showed that the wheat homoeologs have the maximum homology amongst themselves followed by homology to barley. The triticeae (wheat and barley) *AGO4_9 class* genes tend to cluster separately from other related cereals. Particularly, *AGO802* and *AGO1003* form a cluster divergent from all other *AGOs* suggesting a triticeae specific evolution of these genes. The triticeae family is distinctive from related cereals by large genomes; the majority of which are composed of transposons and repetitive DNA [32]. The role of *AGO4_9* genes in silencing of transposons and repetitive DNA is well known [33]. The triticeae-specific *AGO4_9* genes, *AGO802* and *AGO1003* may have a specific role in managing the large genomes of these species.

### *AGO802* is preferentially expressed in embryos during seed development

Argonautes of *AGO4_9* class have been shown to possess an important role in pre- and post-fertilization development. *AGO104* in maize, required for meiosis and knockout mutations in this gene, results in aberrant meiosis leading to the production of unreduced gametes [3]. The *AGO9 class* has been implicated in pre-meiotic megaspore differentiation and also in transposon silencing during gametogenesis in Arabidopsis [2]. Previously, we have shown that the two barley *AGO4_9* genes, *AGO1002* and *AGO1003,* are preferentially expressed during sporogenesis and post-fertilization embryo development [5].

Studies in Arabidopsis have also shown the expression pattern of *AGO9* in reproductive tissues and they have been shown to be necessary for female gamete formation [2]. The authors observed a high expression of *AGO* genes in the pre-fertilization stages, specifically in ovules and anthers, but none in embryos. Another instance where *Pup1* cDNA, corresponding to the *AGO9* gene family, demonstrates an early expression of this gene is found specifically in the tissues of ovules and anthers [34]. To understand the function of *AGO4_9* genes in wheat reproductive development and seed dormancy, we studied their expression in embryos at different stages after fertilization. In general, the relative expression of *AGO802* homoeologs was higher than *AGO804* homoeologs in all of the tissues examined.

Among *AGO802* homoeologs, the expression of *AGO802A* and *AGO802B* was significantly higher than *AGO802D*, which had an expression similar to *AGO804* homoeologs. The two highly expressed genes, *AGO802A* and *AGO802B,* have a converse expression pattern. *AGO802A* has a higher expression in the stages immediately following fertilization, which decreases as the embryo matures. On the other hand, the expression of *AGO804B* is low following fertilization but increases during embryo development reaching a maximum level at 20 DAP and then decreasing during seed maturity and desiccation. In barley, *AGO1003* is expressed at higher levels than *AGO1002* which is consistent in wheat; however, two wheat homoeologs, *AGO802A* and *AGO802B,* appeared to acquire a different pattern of expression. It is possible that the role played by *AGO1003* in barley is assumed by these two homoeologs in wheat, but with a differentiation in function between the early, and late, stages of embryo development.

### *AGO802B* retro-transposon insertion may be related to PHS resistance in wheat

There is increasing evidence that epigenetic changes may play a role in seed dormancy and hence, may determine the PHS reaction of cultivars and varieties. In Arabidopsis *HUB1*, a histone mono-ubiquitation factor is required for seed dormancy and its mutant exhibit reduced seed dormancy [20]. Recently, it has been shown that histone demethylation caused by a mutation in the gene shows increased seed dormancy in Arabidopsis [19]. In barley, the expression of the *AGO1003* gene is reduced in developing embryos in the seeds of PHS resistant varieties [5]. In wheat, we observed that *AGO802B* mimics the expression of *AGO1003* more closely with respect to a role in modulating seed dormancy. Similar to *AGO1003*, the maximum expression of *AGO802B* is in the embryos at 20 DAP and thereafter declines as the seed matures (30 DAP). This stage is particularly important as reprogramming of seed dormancy activates during that time. In wheat, when we analyzed the expression of *AGO802B* in embryos of PHS resistant and susceptible varieties, we observed polymorphism in the size of the amplified region in both the genomic and RT-PCR. The polymorphism is due to the insertion of a retroposon-like DNA sequence, which produced 9 bp terminal end duplication but does not have homology to any known retroposon in the databases.

Interestingly, this insertion was present in six of the seven PHS resistant genotypes and absent in the two PHS susceptible genotypes that were tested. The seven PHS resistant varieties tested were RL4137, Snowbird, AC Karma, AC Domain, AC Vista, Thatcher and SC8021-V2. With the exception of SC8021-V2, all other genotypes have insertion in the *AGO802B* gene. This insertion was also present in the variety CDC Teal which has a medium PHS reaction. Incidentally, the varieties Snowbird, AC Karma, AC Domain, AC Vista, which contain a DNA insertion, are related. These varieties possess RL4137 in their pedigree which is derived from a cross involving Thatcher [35]. It is quite possible that the DNA insertion in the *AGO802B* gene is derived from the Thatcher variety. SC8021-V2 on the other hand was derived from a cross that did not involve either Thatcher or RL4137 [36]. Previous studies in our laboratory suggest a role for DNA methylation in modulating seed dormancy and components of the RdDM pathway, including *AGO1003*, are down regulated in the developing embryos of seed dormant genotypes [5]. The strong correlation of the insertion in *AGO802B* with the PHS resistant genotypes suggests that it may be involved in modulating seed dormancy in these genotypes similar way to the reduced expression of *AGO1003* in barley seeds [5]. The insertion in the *AGO802B* gene is in the 3′ UTR 30 bp downstream of the stop codon and it may have a regulatory effect on this gene. The role of 3′ UTRs in post-transcriptional gene regulation is well known [37]–[38]. It is possible that the insertion in 3′ UTR in *AGO802B* in PHS resistant genotypes has a negative post-translational effect which is similar to transcriptional down regulation of *AGO1003* in barley. This can be verified through western blot analysis if specific antibodies are raised to distinguish not only between the two *AGO4_9 class* genes, but also between the three homoeologs of *AGO802*. However, this may be complicated due to high homology between homoeologs.

In the absence of a suitable tool to assess the differences in post-transcriptional regulation among the different wheat varieties, we tested to determine if there is a change in methylation of DNA repeats in the embryos of mature seeds, which could be indicative of differences in AGO802B activity. *AGO4_9* genes are components of the RdDM pathway which has a role in the methylation of the repetitive DNA, particularly the 5S rDNA and the centromeric repeats [3], [30]. Our observation of methylation pattern at 5S rDNA repeats among six varieties with the variable PHS reaction, indicate that the PHS resistant varieties (RL4137, Snowbird, AC Domain and AC Karma) are hypomethylated at these repeats compared to AC Andrew, a PHS susceptible variety. Consequently, low protein concentration of AGO 4 in PHS resistant varieties is found to be consistent with methylation status (Figure 7).

CDC Teal showed an intermediate methylation status which is consistent with its medium PHS reaction. Further, the methylation pattern is consistent with the polymorphism in *AGO802B* gene suggesting a strong correlation between the DNA insertion, DNA methylation and PHS reaction of wheat varieties. Methylated DNA repeats, such as 5S rDNA, is indicative of a global methylation status and therefore, the above results suggest methylome differences between the embryos of wheat varieties which could underlie the differences in gene activity and their reaction to PHS. In Arabidopsis, histone methylation is also impaired in *ago4–2* mutants, suggesting the close interplay between DNA and histone methylation [29].

RL4137 has been a source of PHS resistance for Canadian wheat and several varieties have been developed using RL4137 as a parent [35]. The PHS resistance of RL4137 has been attributed to two different mechanisms – one associated with the red seed coat color and a second yet unknown mechanism independent of seed coat color [35]. Several white seed coat PHS resistant varieties have been derived from RL4137 although their resistance was not as strong as RL4137 [35], [39]. The pool of PHS resistant varieties investigated in this study had both red and white seed coat color, and all these genotypes suggest that seed color does not have any correlation with polymorphism or methylation status. Our results imply that DNA methylation could be one of the contributors to PHS resistance in RL4137 and its derivatives, which is independent of seed coat colour.

## Materials and Methods

### Plant Material

Plants from ten different wheat varieties with varying degree of seed dormancy along with Chinese Spring (CS) were grown in a growth chamber at 20°C/18°C under a 16-h light/8-h dark cycle. RL4137 [35], Snowbird [7], AC Domain [40], AC Karma [41], SC8021-V2 [36], Thatcher and AC Vista [42] are PHS resistant or tolerant varieties whereas AC Andrew [43] and Sadash [44] are PHS susceptible varieties. The CDC Teal variety has a medium PHS reaction [45]. Samples for RNA extraction were collected at stages corresponding to 5 DAP (days after pollination) for ovaries and 10, 15, 20, 25 and 30 DAP for embryos.

Complete sets of nullisomic and di-telosomic lines were obtained from Wheat Genomics Resource Center (WGRC), Kansas State University and grown in the green house with similar growing conditions as described above. Primers used to map the homoeologs of *AGO802* were AGO802-A, AGO802-B and AGO802-D and for *AGO804* were AGO804-A, AGO804-B and AGO804-D (Table 2).

**Table 2:** List of primers used in mapping studies.

Primer	orientation	Sequence (5′→3′)
AGO802-A	F	CCTGTTGGACTCCTGGGATGTTGT
	R	AGTAAAGGTTCAACTGCAGCACCCTG
AGO802-B	F	CCTGTTGGGACTCCTGGTATGTTGC
	R	AGTAAAGGTTCAACAGCAGCATCCTA
AGO802-D	F	TCCGAGACGTCGTCGAGCCAGGGA
	R	GAAGAACATGCTGCTGCGGACTTT
AGO804-A	F	GAACTGTAGCTGTATGGGTGCCC
	R	AAC TCG ATT ATC CAC CCA AGT CC
AGO804-B	F	GATGGCACCTGGAACTTTAGCTGT
	R	CGCCAACACCACAAACGGTAACAT
AGO804-D	F	AGCTGTATGGGTGGCTGGTAGAAT
	R	AGCTTCAGGTTTGCTTGGCTCAAC

### DNA Extraction and PCR

Genomic DNA was extracted using standard protocols. The PCR reaction consisted of 160 nmol of each primer, 2× TAQ&LOAD PCR master mix (MP Biomedicals) and 20–30 ng DNA in a 20 µl reaction. The PCR conditions were: denaturation at 94°C for 3 min, then 35 cycles at 94°C for 30 s, 58°C for 30 s, 72°C for 30 s and final extension at 72°C for 5 min. The PCR samples were analyzed by electrophoresis on 1% Agarose gel. For sequencing, the amplified products were, either sequenced directly, or cloned in TOPO TA Sequencing vector (Invitrogen). Sequencing services were provided by Genome Quebec.

### RNA extraction and 3′ RACE

Total RNA was extracted from fresh tissue (ovaries or embryos) with TRIAZOL^TM^ (Invitrogen, Burlington, ON) following the manufacturer's recommendations. RNA integrity and concentration were assessed by spectrophotometry with ND1000 (NanoDrop, Wilmington, DE) and the quality of RNA was checked by gel electrophoresis. 3′ RACE was performed using a SMARTerTM RACE cDNA Amplification Kit following the manufacturer's instructions. Briefly, 3′-RACE-Ready cDNA was synthesized in the first step utilizing the 3′-CDS primer A. A total of 500 ng RNA was used in a final volume of 10 μl reverse transcription reaction. cDNA synthesis was followed by PCR amplification using Universal Primer Mix (UPM) and a gene specific primer (GSP). 0.5 μl cDNA from the above reaction was used in 50 μl of PCR reaction with the Advantage™ 2 PCR Enzyme System (Clontech). Nested PCRs were performed using Nested Universal Primer (NUP) and Nested Gene Specific Primer (NGSP). 1 ul of the PCR product from the first round amplification was used in a 50 μl reaction. The PCR program was 94°C for 30 seconds and 72°C for 3 minutes, five cycles; then 94°C for 30 seconds, 70°C for 30 seconds and 72°C for 3 minutes, five cycles; followed by 25 cycles of 94°C for 30 seconds and 68°C for 30 seconds; and concluding with by an extension cycle of 72°C for 3 minutes. The PCR products were subsequently cloned in TOPO TA Sequencing vector (Invitrogen) and sequenced.

### cDNA synthesis and Quantitative RT-PCR

A total of 1 µg RNA was treated with DNase I enzyme (Invitrogen) following the manufacturer's recommendations to remove DNA contamination. 250 ng of DNase treated RNA was reverse transcribed with the iScript cDNA synthesis kit (BioRad, Mississauga, ON). The transcribed cDNA was diluted with an equal amount of sterile water and used for PCR. The integrity of the cDNA was verified by conventional RT-PCR with the *ACTIN* primers followed by gel electrophoresis.

For qRT-PCR, two biological replicates and two technical replicates were performed for each sample and two negative controls were included in each run. QRT-PCR was conducted on the Mx3005 instrument (Agilent Technologies, Cedar Creek, TX) with Briliant III SYBR Green QPCR master mix (Agilent Technologies) following the manufacturer's recommendations. Amplification was performed in a 20 µL reaction mixture containing 160 nmol each primer, 1× Briliant III SYBR Green QPCR master mix, 15 µM ROX reference dye and 1 µL of cDNA template. The amplification conditions were 95°C for 10 min (hot start), followed by 30 cycles at 94°C for 30 s, 58°C for 30 s, 72°C for 30 s. The fluorescence reading was taken at 72°C at the end of the elongation cycle. The relative expression ratios of the target genes versus reference genes were calculated with the equation developed by Zhao and Fernald [46] based on crossing point (CP) and efficiency obtained for each sample amplified with the reference genes and the target genes using the equation: R0 = 1/(1+E)ˆCT where R0 is the initial template concentration, E is the efficiency in the exponential phase and CT is the cycle number at threshold. Analysis was performed using web-based software – Realtime PCR Miner (http://www.miner.ewindup.info/version2).

### Bioinformatics and sequence analysis

Phylogenetic analysis and protein sequence alignments were done using Biology Workbench (http://workbench.sdsc.edu/) and Clone Manager9 software respectively. Primers for qRT-PCR analysis were designed using IDT PrimerQuest software (http://www.idtdna.com/scitools/applications/primerquest/). Primers sequences used were –1002F: 5′TGGGTGCCCGTAGAATGAACAA-GT3′and 1002R: 5′GGTTGGTTGGTTGGTTCAACGAC3′for *AGO1002*; 1003F: 5′GAAAGTCC-GCAGCAGCATGTTCTT3′ and 1003R: GTGGCAGCAAGCTTAGGAACACAA for *AGO1003*. *ACTIN* was used as a reference gene using the primers from [47].

### DNA Gel Blot Analysis

To analyze the methylation of 5S rDNA repeats 15 ug DNA was digested with MspI restriction enzyme, electrophoretically separated on 0.8% agarose gels, transferred to Hybond N^+^ nylon membrane (Amersham Pharmacia Biotech, Piscataway, NJ, USA), hybridized with ^32^P-labeled barley 5S rDNA probe and washed according to manufacturer's instructions. 5S rDNA probe was obtained through amplification from barley genomic DNA with 5S primers (Hv5S-F: CGAAGTTAAGCGTGCTTGG and Hv5S-R: GCATCCGACATGCAACTATC). Amplified products were cloned and sequenced to verify the correct amplification. Labeling of the probe was performed according to manufacturer's instructions, using Ready-To-Go^TM^ DNA Labeling Beads (Amersham Bioscience, Buckinghamshire, England, UK).

### Protein extraction and immunoblotting

Proteins were extracted from embryos of six wheat varieties using modified protocol of Singh et al. [48]. One gram of grinded embryos were mixed with 2 ml of a buffer (pH 7.4) containing 50 mM 2-amino-2-(hydroxymethyl)-1,3-propanediol (TRIS)–HCl, 200 mM NaCl, 10 mM, CaCl_2_, 0.1% triton and 1% EDTA-free protease inhibitor. The extract was centrifuged at 14000 rpm and supernatant was collected for further analysis. Equal amounts of protein were loaded on 8% SDS- PAGE gels. Proteins were transferred to a PVDF membrane, blocked in 5% skim milk powder and incubated with Anti-AGO4 antibody (Agrisera AB, Vännas, Sweden). After incubation with HRP-conjugated secondary antibody, chemiluminescence detection was performed [49] using Clarity^tm^ Western ECL substrate and ChemiDoc™ XRS+ system (Biorad, Hercules, CA, USA).
