# Correction: Polymorphic Homoeolog of Key Gene of RdDM Pathway, *ARGONAUTE4_9 class* Is Associated with Pre-Harvest Sprouting in Wheat (*Triticum aestivum* L.)

**DOI:** 10.1371/journal.pone.0106986

**Published:** 2014-08-22

**Authors:** The PLOS ONE Staff

The map locations of the two wheat *AGO4_9* class genes *AGO802* and *AGO804* are incorrect throughout the article. The correct map locations should be chromosomes 3L and 1L.

The seventh sentence of the Abstract is incorrect. It should read: Our results indicate that the two wheat *AGO4_9* class genes i.e. *AGO802* and *AGO804* map to chromosomes 3L and 1L are preferentially expressed in the embryos of developing seeds.

The heading of the third subsection in the Results is incorrect. It should read: *AGO802* and *AGO804* map to chromosome 3L and 1L.

There is an error in the fifth sentence of the Discussion. It should read: *AGO802* maps to chromosome 3L whereas *AGO804* maps to chromosome 1L based on the homoeolog-specific PCR primers.

There are errors in the legend for Figure 3, “Mapping of *AGO802* and *AGO804* in wheat.” The complete, correct Figure 3 legend is:

**Figure 3:**
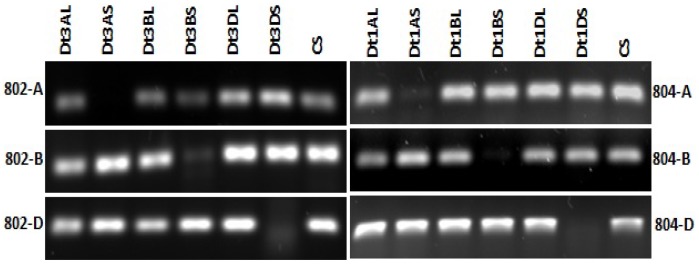
Mapping of *AGO802* and *AGO804* in wheat. Absence of amplification in lanes Dt3AS, Dt3BS and Dt3DS in *AGO802*, suggest its map location on chromosome arm 3L. While in *AGO804*, absence of amplification in Dt1AS, Dt1BS and Dt1DS lanes, confirms its map location on chromosome 1L.
